# EXPLORING MECHANISMS OF AGING THROUGH LONGITUDINAL TRAJECTORIES: BIOLOGICAL, PHENOTYPIC, AND CLINICAL

**DOI:** 10.1093/geroni/igz038.2144

**Published:** 2019-11-08

**Authors:** Jennifer A Schrack, Luigi Ferrucci

**Affiliations:** 1 Johns Hopkins University, Baltimore, Maryland, United States; 2 Translational Gerontology Branch, Intramural Research Program, National Institute on Aging, National Institutes of Health, Baltimore, Maryland, United States

## Abstract

Over the past several decades, researchers have searched for early and accurate predictors of healthy aging, establishing metrics to potentially quantify the aging process based on functional performance, disease diagnosis, or other clinical tests. Using these metrics, efforts have been made to disentangle the concepts of “normal” versus “accelerated” aging to identify individuals transitioning into states of disease and disability, allowing for more targeted and effective treatment(s). Yet, many of the variables included in these indices have been chosen based on their availability using cross-sectional associations with chronological age. Such variables may reflect birth cohort differences and selective attrition and fail to accurately represent the mechanisms most representative of the aging process. Using data longitudinal data from the Baltimore Longitudinal Study of Aging and beyond, this symposium will present a conceptual framework of phenotypic aging and its relationship with biological aging, and trajectories of aging phenotypes using state-of-the-art measures collected over the past 10 years. Specifically, we will discuss new insights into trajectories of molecular (inflammatory and metabolic markers), physiological (energetics, body composition, brain atrophy), and clinical measures (gait speed and executive function), and discuss methodological considerations for combining these phenotypes into a measure that can be linked with biological aging measured using a systems approach to improve and refine future understanding of mechanisms of “normal” versus “accelerated” aging. Further development and application of the methodology presented in this symposium will highlight the importance of using longitudinal data to improve understanding of physical and cognitive trajectories with aging.

